# Experimental Study on Chemical–Mechanical Synergistic Preparation for Cemented Carbide Insert Cutting Edge

**DOI:** 10.3390/mi15010017

**Published:** 2023-12-21

**Authors:** Changjiang Qin, Jian Pan, Lei Guo, Chi Zhang, Wanli Chen, Zihua Hu, Shengqiang Jiang, Xiaogao Chen, Meijiao Mao

**Affiliations:** School of Mechanical Engineering and Mechanics, Xiangtan University, Xiangtan 411105, China; panjian802@163.com (J.P.); 13551757978@163.com (L.G.); m19976601099@163.com (C.Z.); a15623412023@163.com (W.C.); iamtxtu@163.com (Z.H.); jsqcx@126.com (S.J.); 17769498801@163.com (X.C.); maomj2000@163.com (M.M.)

**Keywords:** cemented carbide insert, chemical–mechanical synergistic preparation (CMSP), cutting edge, material removal rate (MRR), surface roughness

## Abstract

Typical edge defects in the edge region of a new cemented carbide insert without edge preparation include burrs, poor surface quality, micro-breakages, and irregularities along the edge. To address the problems in new cemented carbide inserts without edge preparations, a chemical–mechanical synergistic preparation (CMSP) method for the cemented carbide insert cutting edge was proposed. Firstly, the CMSP device for the insert cutting edge was constructed. Then, the polishing slurry of the CMSP for the insert cutting edge was optimized using the Taguchi method combined with a grey relation analysis and fuzzy inference. Finally, orthogonal experiments, the Taguchi method, and analysis of variance (ANOVA) were used to investigate the effect of the polishing plate’s rotational speed, swing angle, and input frequency of the controller on the edge preparation process, and the parameters were optimized. The results showed that the best parameter combination for the polishing slurry for the cemented carbide inserts was the mass concentration of the abrasive particle of 10 wt%, the mass concentration of the oxidant of 10 wt%, the mass concentration of the dispersant of 2 wt%, and the pH of 8. The CMSP process parameter combination for the linear edge had the polishing plate’s rotational speed of 90 rpm, the swing angle of 6°, and the input frequency of the controller of 5000 Hz. The optimum CMSP process parameter combination for the circular edge had the polishing plate’s rotational speed of 90 rpm, the swing angle of 6°, and the input frequency of the controller of 7000 Hz. The polishing plate’s rotational speed had the most significant impact on the edge preparation process, followed by the swing angle, and the effect of the input frequency of the controller was the smallest. This study demonstrated that CMSP is a potential way to treat the cemented carbide insert cutting edge in a tool enterprise.

## 1. Introduction

Due to significant hardness, wear resistance, strength, and toughness, cemented carbide inserts have broad applications in cutting difficult-to-machine materials such as stainless steel, titanium alloys, and superalloys [1]. To meet the precision machining requirements of high contour accuracy and high surface quality for the parts, stable ultra-precision machine tools, process parameters, and machining environments are required [2,3], but a high-quality cemented carbide insert is one of the key technologies. In the machining process, the cutting contact region between a carbide insert and a part includes the rake face, the flank face, and the circular arc or linear region of the cutting edge. The cutting edge is responsible for the main task of material removal. The geometrical parameters and mechanical properties of a cutting edge directly affect the stability of the machining process and the machined surface’s roughness [4]. Therefore, it is required that the surface integrity of a cemented carbide insert cutting edge is good, there is no surface/sub-surface damage layer, stable cutting can be carried out, and the tool life can be improved. Therefore, production costs can be reduced and material utilization can be improved. The surface of a new, sharp cemented carbide insert without an edge preparation is machined with diamond grinding in various conditions, and there are typical edge defects in the edge region, such as micro-breakages, burrs, bad surface quality, and irregularities along the edge. To reduce and eliminate edge defects and improve the cutting performance of an insert, cutting edge preparation is carried out by tool manufacturers after the final grinding process of an insert manufacturing process is completed. Many studies have been conducted on cemented carbide insert cutting edge preparation methods. Denkena et al. [5] proposed a five-axis brushing technique for preparing a carbide insert edge and found that an insert with edge preparation could improve the machined surface quality of a part. Yussefian et al. [6] used electrical spark discharges to prepare a cemented carbide insert edge and found that an insert with edge preparation could improve tool life. Zhang et al. [7] used a wet micro-abrasive blasting method to prepare a carbide insert edge, and this method could improve tool life and cutting performance. Wang et al. [8] used a pressurized air wet abrasive jet machining method to prepare a cemented carbide insert cutting edge, and it was found that an insert with edge preparation could improve tool life and achieve better part surface quality. Wang et al. [9] used wet abrasive jet machining, brushing, and drag finishing methods to prepare cemented carbide insert edges, and they found that the residual compressive stress levels of the edges prepared with abrasive jet machining were 63% higher than those of the edges without preparation, and the surface roughness levels of the edges prepared with drag-finishing were the lowest, while the cutting performances of the edges prepared with drag finishing were also the best. Zimmermann et al. [10] used laser machining to prepare the cutting edge of a cemented carbide tool and found that the formation of residual tensile stresses and the deterioration of the mechanical properties of the surface layer of a tool could be reduced with appropriate laser-machining parameters. 

To obtain a better surface quality of an insert cutting edge, some scholars have proposed a polishing method to prepare the edges of carbide inserts. Lyu et al. [11] proposed a brush-assisted shear-thickening polishing method for the preparation of a cemented carbide insert cutting edge, and this method could obtain better surface quality of a cutting edge. Shao et al. [12] employed a flexible fiber-assisted shear-thickening polishing method for the preparation of a carbide insert cutting edge and studied the effect of the polishing angle and polishing speed on the efficiency of the cutting edge preparation. This method could also obtain a better surface quality of a cutting edge.

The chemical–mechanical polishing (CMP) method is a widely recognized ultra-precision machining method with global planarization at the nanoscale [13]. This method mainly relies on the mechanical action of abrasive particles and the chemical action of oxidants in a polishing slurry to make the surface of a polished workpiece flat and smooth, which has the advantages of no surface/subsurface damage, low density of surface defects, and low cost [14]. Moreover, CMP technology has been employed for polishing the rake faces of carbide inserts, and it has been found that this method can improve the surface quality and cutting performance of an insert [15,16]. In addition, Qin et al. [17] investigated the material removal mechanism of the chemical mechanical polishing of a carbide insert and established a material removal model for the chemical mechanical polishing of a carbide insert.

Because of the advantages of CMP technology, the chemical–mechanical synergistic preparation (CMSP) of the cemented carbide insert cutting edge was proposed to improve the surface integrity of the cemented carbide insert edge. Firstly, the device for the CMSP of cemented carbide insert cutting edges was constructed. Then, the polishing slurry for the CMSP of the cemented carbide insert cutting edge was optimized using the Taguchi method combined with a grey relation analysis and fuzzy inference. Finally, orthogonal experiments, range analysis, and analysis of variance (ANOVA) were used to investigate the effects of the polishing plate’s rotational speed of the swing angle and input frequency of the controller on the edge preparation process, and the edge preparation process parameters were optimized.

## 2. Construction of CMSP Platform

**Figure 1:**
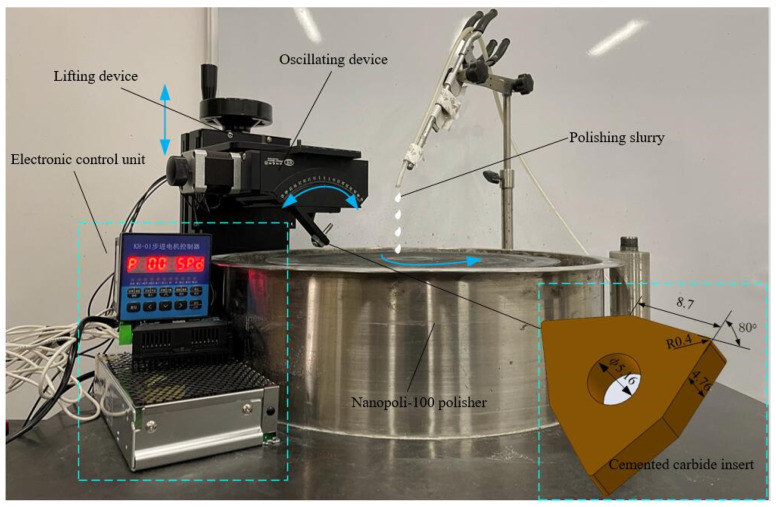
The CMSP platform.

The CMSP platform is composed of a Nanopoli-100 polisher (Hangzhou Zhibang Nanotechnology Co., Ltd., Hangzhou, China), a lifting device, a fixture, an oscillating device, and an electric control device, as shown in Figure 1. The insert is fixed on the fixture with a screw. The fixture is connected to the oscillating device with the screws. The oscillating device is connected to the lifting device with the screws. The lifting device is mainly composed of a ball screw pair. The lifting device is adjusted to bring the insert edge in contact with the polishing pad and apply a certain polishing pressure. The polishing slurry is supplied by a peristaltic pump to drip onto the polishing pad at a certain flow rate. Meanwhile, the oscillating device is controlled by an electronic control device to swing the insert edge periodically within a certain angle range. The polishing slurry continuously flows through the insert edge with the rotation of the polishing pad. The oxidant in the polishing slurry reacts with the cutting edge to form a layer of oxide film. At the same time, the oxide film is removed by the mechanical action of the abrasive particles in the polishing slurry. The formation and removal of the film are alternated continuously. Therefore, the cutting edge preparation can be realized.

## 3. Methods and Experiments

### 3.1. The Polishing Slurry Optimization Experiment

**Figure 2:**
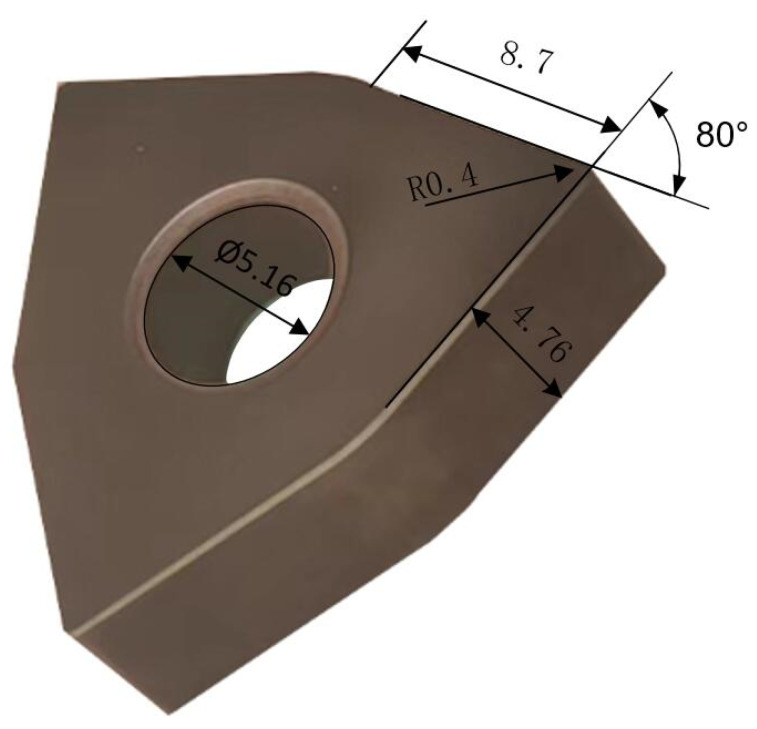
YG8 insert.

To achieve efficient and low-cost preparation of the carbide insert edge, the Taguchi method, combined with the grey relational analysis and fuzzy inference, is applied to optimize the CMSP polishing slurry for the cemented carbide inserts. The polishing object is a YG8 cemented carbide insert (Zhuzhou Cemented Carbide Cutting Tools Co., Ltd., Zhuzhou, China), as shown in Figure 2. Through the previous CMP experiments, the alumina abrasive particles are used as the abrasive particles, and the oxidant is H_2_O_2_ for the cemented carbide insert CMP, which can achieve high MRR and better surface quality [18]. In this study, the alumina abrasive particle with a particle size of 500 nm is chosen as the polishing abrasive particle. The oxidant is H_2_O_2_. The sodium dodecyl sulfate (SDS) is selected as the dispersant. Dilute sulfuric acid (H_2_SO_4_) and sodium hydroxide (NaOH) are used as pH regulators. The pH value of the slurry is measured using a digital PHS-3C Benchtop pH meter (Shaoxing Supo Instrument Co., Ltd., Shaoxing, China). Considering the dispersibility of the abrasive particles and the pH value of the polishing slurry on the MRR and surface roughness Ra, the abrasive particle concentration, oxidant concentration, dispersant concentration, and the pH value of the polishing slurry are selected as the main factors. The CMP orthogonal experiment for the insert is designed as a 4-factor 3-level Taguchi L9 orthogonal array, as shown in Table 1. The polishing experiment parameters are listed in Table 2. The polishing slurry is prepared according to Table 1. Then, the CMP experiment of the insert rake face is carried out on the Nanpoli-100 polisher. The polishing pad material is a porous polyurethane. A peristaltic pump supplies the polishing slurry with an adjustable flow rate. After the CMP of the inserts is finished, the inserts are cleaned in an ultrasonic cleaner with acetone for 5 min, then with anhydrous alcohol for 3 min, and finally with deionized water for 2 min. The stylus type surface roughness measuring instrument (JB-5C, Shanghai Taiming Optical Instrument Co., Ltd., Shanghai, China) with an accuracy of 0.001 μm is applied to measure the surface roughness Ra of the polished inserts three times, and the average value is regarded as the experimental data. Then, an electronic balance with an accuracy of 1/10,000 g is used to measure the weight of the inserts three times, and the average value is regarded as experimental data. The MRR of CMP for the cemented carbide insert is estimated as

(1) $$MRR=\frac{\Delta M}{\rho \times S\times t},$$

where ΔM is the difference in insert weight before and after polishing, ρ is the density of the insert, S is the contact area between the insert and the polishing pad, and t is the polishing time.

### 3.2. Cutting Edge Preparation Experiment

According to the optimal polishing slurry obtained in Section 3.1, the effect of the polishing plate’s rotational speed, swing angle, and input frequency of the controller on the edge preparation process are investigated based on the CMSP platform. The polishing plate’s rotational speed, swing angle, and input frequency of the controller are selected as the main factors. The orthogonal experiment of the edge preparation for the cemented carbide inserts is designed as a 3-factor 3-level Taguchi L9 orthogonal array, as shown in Table 3.

The YG8 cemented carbide inserts are selected for the preparation experiment. The preparation parameters are listed in Table 4. 

**Figure 3:**
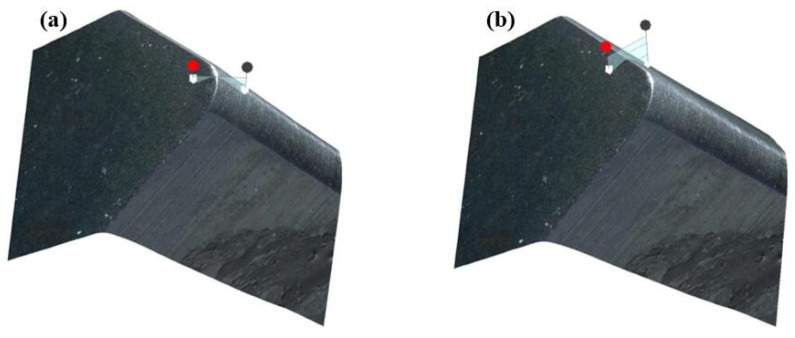
The surface perpendicular to the edge constructed in the VHX-2000 ultra depth of field microscope system: (**a**) circular edge, (**b**) linear edge.

The edge sharpness of the inserts after CMSP can be characterized by the cutting edge radius. Specifically, the cross-section intersection interception function in the VHX-2000 ultra-depth of field microscope system (KEYENCE, Tokyo, Japan) is used to construct a surface perpendicular to the edge, as shown in Figure 3. Then, the edge radius of the insert is obtained by sequentially selecting more than 30 points of the arc of the edge for the least squares fitting and calculation based on Matlab 2022. Taking the insert edge radius as the evaluation index, the edge preparation parameters are optimized by the Taguchi method. The effect of the polishing plate’s rotational speed, swing angle, and input frequency of the controller on the edge preparation process are investigated by using ANOVA. The cutting edge morphology before and after preparation is observed by the VHX-2000 ultra-depth of field microscope and SEM.

## 4. Results and Discussions

### 4.1. The Optimization of the Polishing Slurry

#### 4.1.1. The Calculation of the S/N Ratio of MRR and Surface Roughness Ra

The polishing slurry is prepared according to Table 1, and then the polishing experiments are carried out. The MRR and surface roughness Ra of the YG8 insert are obtained, as shown in Table 5.

In this study, it is expected that the MRR of the CMP for the cemented carbide insert should be as large as possible, and the surface roughness Ra of the polished cemented carbide insert should be as low as possible. Therefore, the larger the better case of the Taguchi method is applied to calculate the S/N ratio of the MRR, and the smaller-the-better case of the Taguchi method is used to calculate the S/N ratio of the surface roughness Ra, which can be expressed as follows:

(2) $$S/N\;\mathrm{ratio}=-10\cdot \log\left[\frac{1}{n}\cdot \sum_{i=1}^{n}\frac{1}{y_{i}^{2}}\right],$$

(3) $$S/N\;\mathrm{ratio}=-10\cdot \log\left[\frac{1}{n}\cdot \sum_{i=1}^{n}y_{i}^{2}\right],$$

where $y_{i}$ is the experimental value; and n denotes the number of experiments. Calculation results are listed in Table 6.

#### 4.1.2. The Grey Rational Analysis 

In the grey relational analysis method, when the range of the sequence is too large, the role of some factors is often ignored. When the target directions of each factor in the sequence are inconsistent, grey relational analysis may also result in wrong results. Firstly, it is necessary to perform data pre-processing for the entire sequence. The initial sequence is converted into a comparable sequence with the data pre-processing. The conversion is called grey relational generation. The initial sequence data are normalized in the range between zero and one. In this study, it is expected that the S/N ratio of the MRR and the surface roughness Ra should be as large as possible. The initial sequence data (S/N ratio of the MRR and the surface roughness Ra) are normalized with the “larger-the-better” characteristic of a linear data pre-processing method, which can be expressed as

(4) $$x_{i}(k)=\frac{m_{i}(k)-\min m_{i}(k)}{\max m_{i}(k)-\min m_{i}(k)},$$

where $x_{i}(k)$ is the value after data pre-processing, $m_{i}(k)$ denotes the initial sequence data, $\min m_{i}(k)$ denotes the minimum value of $m_{i}(k)$ , $\max m_{i}(k)$ denotes the maximum value of $m_{i}(k)$ . The normalized S/N ratios of MRR and surface roughness Ra are listed in Table 7.

Then, the grey rational coefficient $n_{ij}(k)$ is calculated based on the normalized S/N ratios of MRR and surface roughness Ra, which can be expressed as

(5) $$n_{ij}(k)=\frac{\Delta_{\min}+\xi \Delta_{\max}}{\Delta_{ij}(k)+\xi \Delta_{\max}},$$

where $\Delta_{ij}(k)=\left\|x_{i}(k)-x_{j}(k)\right\|$ denotes the deviation in absolute value between *x_i_*(*k*) and *x_j_*(*k*), *x_j_*(*k*) = 1(*j* = 1, 2, …, 16) is the comparable sequence, ξ is the distinguishing coefficient, $\Delta_{\min}=\overset{\min}{\forall }j\in i\overset{\min}{\forall }k\left\|x_{i}(k)-x_{j}(k)\right\|$ is the minimum value of $\Delta_{ij}(k)$ , $\Delta_{\max}=\overset{\max}{\forall }j\in i\overset{\max}{\forall }k\left\|x_{i}(k)-x_{j}(k)\right\|$ is the maximum value of $\Delta_{ij}(k)$ . The grey rational coefficients of the MRR and Ra are listed in Table 8.

#### 4.1.3. Fuzzy Inference

The inference system based on fuzzy rules consists of three basic units: fuzzier, fuzzy inference engine, and defuzzifier. The input vector of the gray relational coefficients for MRR and the surface roughness Ra is first fuzzified by the fuzzifier using the membership functions. Then, the fuzzy inference is performed by the fuzzy inference engine using the fuzzy rules to generate the fuzzy values. Finally, the fuzzy values are transformed to the multi-performance characteristics index (MPCI). In this study, the gray relational coefficients of MRR and the surface roughness Ra are regarded as the input variable for the fuzzy inference system (FIS), respectively. The MPCI value is regarded as the output variable. Based on MATLAB applications, the FIS architecture is established. The triangular membership function is chosen for the input variable. There are three membership functions, named small (S), middle (M), and large (L), respectively. The triangular membership function is selected for the output variable MPCI, and there are five membership functions, named very small (VS), small (S), middle (M), large (L), and very large (VL), respectively. Therefore, nine fuzzy rules are generated in the rule editor of MATLAB, that is,

$$\begin{array}{l} Rule\;1:\;if\;m\;is\;S\;and\;r\;is\;S\;then\;y\;is\;VS;\\ Rule\;2:\;if\;m\;is\;S\;and\;r\;is\;M\;then\;y\;is\;S;\\ Rule\;3:\;if\;m\;is\;S\;and\;r\;is\;L\;then\;y\;is\;M;\\ Rule\;4:\;if\;m\;is\;M\;and\;r\;is\;S\;then\;y\;is\;S;\\ Rule\;5:\;if\;m\;is\;M\;and\;r\;is\;M\;then\;y\;is\;M;\\ Rule\;6:\;if\;m\;is\;M\;and\;r\;is\;L\;then\;y\;is\;L;\\ Rule\;7:\;if\;m\;is\;L\;and\;r\;is\;S\;then\;y\;is\;M;\\ Rule\;8:\;if\;m\;is\;L\;and\;r\;is\;M\;then\;y\;is\;L;\\ Rule\;9:\;if\;m\;is\;L\;and\;r\;is\;L\;then\;y\;is\;VL; \end{array}$$

where y is the output, m and r are the gray relational coefficients inputs for MRR and surface roughness Ra, respectively. Then, the MPCI of each parameter combination is obtained based on the FIS of Matlab, as listed in Table 9.

#### 4.1.4. The Optimization of Polishing Slurry

**Figure 4:**
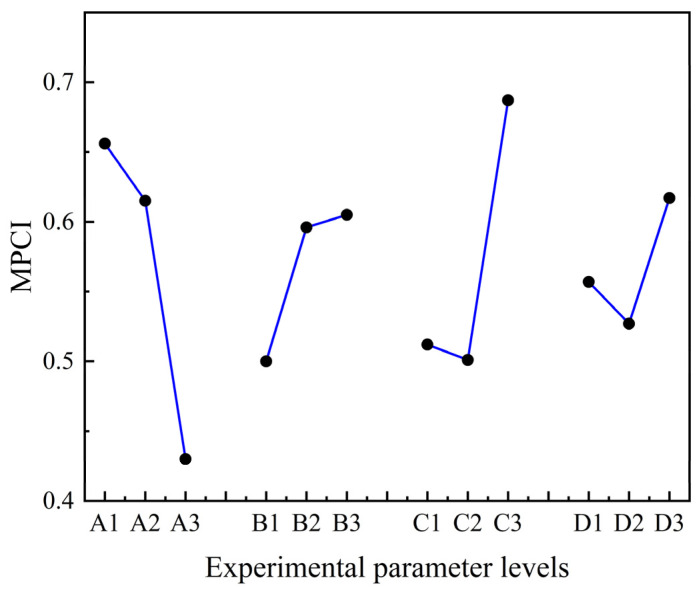
The MPCI values for each factor at their corresponding levels.

To obtain the overall optimal parameter combination of polishing slurry, combining Table 5 with Table 9, the MPCI response for each factor at their corresponding levels is obtained, as shown in Figure 4.

**Figure 5:**
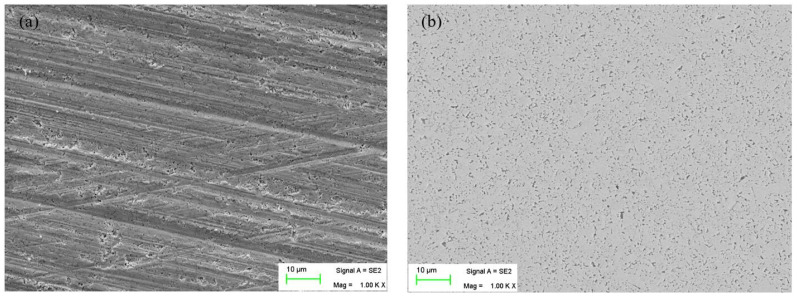
The insert rake surface morphology under SEM: (**a**) before polishing, (**b**) after polishing.

In Figure 4, it can be seen that A1B3C3D3 is the best parameter combination of polishing slurry for cemented carbide inserts. Namely, the best parameter combination of polishing slurry for cemented carbide inserts is with the mass concentration of the abrasive particle of 10 wt%, the mass concentration of the oxidant of 10 wt%, the mass concentration of the dispersant of 2 wt%, and a pH of 8. As the concentration of abrasive particles increases, the MPCI also increases. The reason is that as the concentration of abrasive particles increases, the number of effective abrasive particles involved in material removal increases, leading to an increase in MRR and a decrease in surface roughness Ra. Furthermore, as the concentration of the oxidant increases, the MPCI also increases. The reason is that as the concentration of oxidant increases, the chemical reaction between the surface of the cemented carbide insert and the oxidant is promoted, and MRR increases. The abrasive particles tend to aggregate and settle in the polishing slurry, and have an impact on MRR and surface quality. The abrasive particles can be dispersed by the dispersant without affecting the thermal conductivity of the polishing slurry, thereby ensuring the high dispersion stability of the polishing slurry. As the concentration of dispersant increases, MPCI first decreases and then slightly increases. The reason is that when the dispersant dosage is too high, the molecular chains of unabsorbed dispersant in the solvent tend to entangle with each other due to the supersaturated adsorption on the surface of the alumina particles, leading to depletion flocculation and agglomeration of abrasive particles, thereby affecting MRR and surface quality [18]. The pH of the suspension has a significant impact on the dispersion of the polishing slurry and the chemical reaction between the insert surface and the oxidant [19], thereby affecting the MRR and surface quality. When the pH value is eight, the MPCI reaches the maximum value. It indicates that when the pH value is eight, the dispersion of the polishing slurry and the chemical reaction between the insert surface and the oxidant may be in a good state. Compared with the initial parameter combination A3B3C2D1, MRR is increased by 163%, and the surface roughness Ra is reduced by 40%. Then, the insert rake surface morphology before and after polishing with the best parameter combination of polishing slurry is observed by SEM, as shown in Figure 5.

In Figure 5, it can be seen that there is a lot of scratches on the insert rake surface before polishing. However, after the insert rake surface is polished with the best parameter combination of polishing slurry, the scratches are completely removed, and the rake surface is smooth. So the best parameter combination of polishing slurry is effective and can obtain a smooth surface.

### 4.2. Comprehensive Analysis of CMSP for the Cemented Carbide Insert Edge

According to Table 3, the orthogonal experiment of CMSP for the cemented carbide insert cutting edge is carried out. Then, the edge radius of the insert is obtained by the least squares fitting and calculation based on Matlab, which are listed in Table 10.

**Figure 6:**
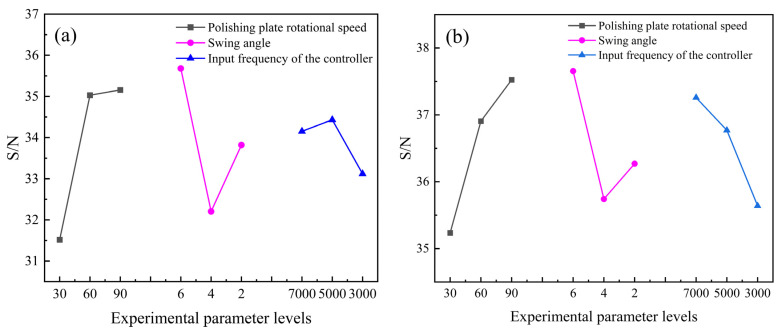
The average response of S/N for each factor at their corresponding levels: (**a**) linear edge, (**b**) circular edge.

It is desirable that the edge radius should be as large as possible in the same preparation time. So the larger the better case of the Taguchi method is applied to calculate the S/N ratio of the linear edge radius and circular edge radius, which are listed in Table 11. Figure 6 shows the average response of S/N for each factor at their corresponding levels.

It is noted in Figure 6 that the best combination of CMSP parameters for the linear edge and circular edge is determined to be A3B1C2 and A3B1C1, respectively. Namely, the best CMSP process parameter combination for the linear edge is with the polishing plate’s rotational speed of 90 rpm, the swing angle of 6°, and the input frequency of the controller of 5000 Hz, and the best CMSP process parameter combination for the circular edge is with the polishing plate’s rotational speed of 90 rpm, the swing angle of 6°, and the input frequency of the controller of 7000 Hz.

To explore the effect of the polishing plate’s rotational speed, swing angle, and input frequency of the controller on the cutting edge radius, based on Table 10, ANOVA results of CMSP for the insert linear and circular edge radius are shown in Table 12 and Table 13, respectively.

From Table 12 and Table 13, it can be seen that the polishing plate’s rotational speed plays a predominant role in the linear edge and circular edge preparation, followed by the swing angle and the input frequency of the controller.

**Figure 7:**
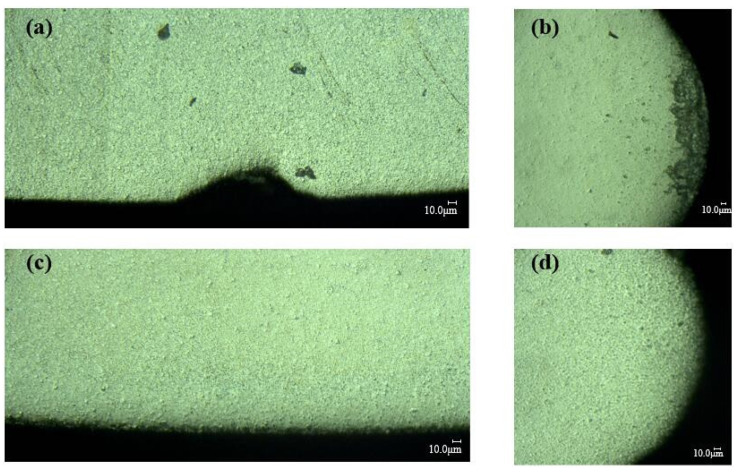
The morphology of the cutting edge under the VHX-2000 ultra depth of field microscope: (**a**) linear edge before the preparation, (**b**) circular edge before the preparation, (**c**) linear edge after the preparation, (**d**) circular edge after the preparation.

**Figure 8:**
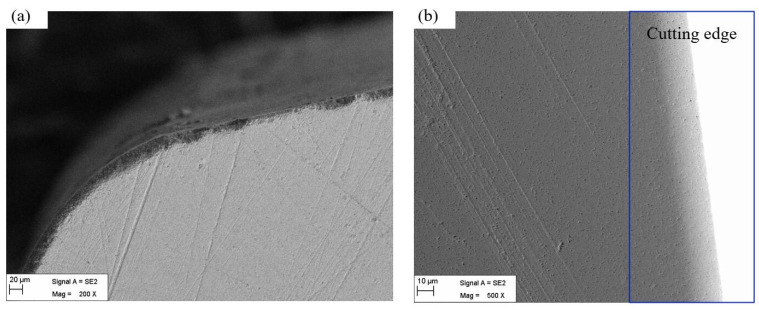
The morphology of the cutting edge under SEM: (**a**) cutting edge before the preparation, (**b**) cutting edge after the preparation.

The cutting edge is prepared with the best combination of CMSP parameters for the linear edge and circular edge of the insert. Then, the cutting edge surface morphology before and after preparation is observed by the VHX-2000 ultra depth of field microscope and SEM, as shown in Figure 7 and Figure 8.

In Figure 7 and Figure 8, it can be seen that the initial cutting edge has some defects in the edge region such as burrs, poor surface finish, micro-breakages, and irregularities along the edge. After the cutting edge is prepared with the best combination of CMSP parameters, the defects are completely removed, and the cutting edge is smooth. So CMSP can improve the surface quality of the cutting edge. Therefore, in the actual cutting process, it can effectively reduce tool wear, improve the machining process stability, and reduce the machined surface quality.

## 5. Conclusions

In this work, the CMSP device for the cemented carbide insert cutting edges is constructed. Then, the polishing slurry of CMSP for cemented carbide insert cutting edge is optimized using the Taguchi method coupled with the grey relational analysis and fuzzy inference. Finally, the orthogonal experiments, the Taguchi method, and ANOVA are used to investigate the effect of the polishing plate’s rotational speed, swing angle, and input frequency of the controller on the edge preparation process, and the parameters are optimized. Thus, the following conclusions can be drawn:

1. A1B3C3D3 is the optimal parameter combination of polishing slurry for cemented carbide inserts. Namely, the best parameter combination of polishing slurry for cemented carbide inserts is with the mass concentration of the abrasive particle of 10 wt%, the mass concentration of the oxidant of 10 wt%, the mass concentration of the dispersant of 2 wt%, and the pH of 8. Compared with the initial parameter combination, A3B3C2D1, the MRR is increased by 163%, and the surface roughness Ra is reduced by 40%.
2. The best combination of CMSP parameters for the linear edge and circular edge is determined to be A3B1C2 and A3B1C1, respectively. Namely, the best CMSP process parameter combination for the linear edge is with the polishing plate’s rotational speed of 90 rpm, the swing angle of 6°, and the input frequency of the controller of 5000 Hz, and the optimum CMSP process parameter combination for the circular edge is with the polishing plate’s rotational speed of 90 rpm, the swing angle of 6°, and the input frequency of the controller of 7000 Hz.
3. The polishing plate’s rotational speed plays a predominant role in linear edge and circular edge preparation, followed by the swing angle and the input frequency of the controller.
4. The CMSP method can not only achieve cutting edge preparation, but also improve the surface quality of the cemented carbide insert cutting edge. In the future, extensive research is required to elucidate the material removal mechanism of CMSP for the cemented carbide insert cutting edge.

## Figures and Tables

**Table 1:** The CMP orthogonal experiment for the insert.

No.	A, The Mass Concentration of Abrasive Particle (wt%)	B, The Mass Concentration of Oxidant (wt%)	C, The Mass Concentration of Dispersant (wt%)	D, pH
1	10	6	4	11
2	10	8	3	9.5
3	10	10	2	8
4	8	6	3	8
5	8	8	2	11
6	8	10	4	9.5
7	6	6	2	9.5
8	6	8	4	8
9	6	10	3	11

**Table 2:** CMP experiment parameters.

Parameter	Value
Polishing pressure (kPa)	94.99
Polishing plate’s rotational speed (rpm)	70
Polishing time (min)	30
The flow rate of polishing slurry (mL/min)	6
Polishing slurry temperature (°C)	25

**Table 3:** The orthogonal experiment scheme of CMSP for the cemented carbide inserts cutting edge.

No.	A, Polishing Plate’s Rotating Speed (rpm)	B, Swing Angle (°)	C, Input Frequency of the Controller (Hz)
1	30	6	7000
2	30	4	5000
3	30	2	3000
4	60	6	5000
5	60	4	3000
6	60	2	7000
7	90	6	3000
8	90	4	7000
9	90	2	5000

**Table 4:** The parameters of CMSP for the cemented carbide insert cutting edge.

Parameters	Value
Polishing pressure (N)	5.5 (Linear cutting edge), 6 (Circular cutting edge)
Polishing slurry flow rate (mL/min)	4
Polishing slurry temperature (°C)	25
Preparation time (min)	5

**Table 5:** The experimental results of the MRR and surface roughness Ra of the insert.

No.	A, The Mass Concentration of Abrasive Particle (wt%)	B, The Mass Concentration of Oxidant (wt%)	C, The Mass Concentration of Dispersant (wt%)	D, pH	MRR (nm/min)	Surface Roughness Ra (nm)
1	10	6	4	11	66.85	47.33
2	10	8	3	9.5	74.81	47.5
3	10	10	2	8	79.58	27.83
4	8	6	3	8	39.79	31.5
5	8	8	2	11	55.71	27.33
6	8	10	4	9.5	57.3	35.5
7	6	6	2	9.5	41.38	40.17
8	6	8	4	8	50.93	41.33
9	6	10	3	11	30.24	46.5

**Table 6:** The S/N ratio of the MRR and surface roughness Ra.

No.	S/N Ratio of the MRR (dB)	S/N Ratio of the Surface Roughness Ra (dB)
1	36.5020	−33.5033
2	37.4791	−33.5339
3	38.0165	−28.8912
4	31.9959	−29.9662
5	34.9185	−28.7337
6	35.1631	−31.0046
7	32.3364	−32.0774
8	34.1402	−32.3259
9	29.6122	−33.3491

**Table 7:** The normalized S/N ratios of MRR and surface roughness Ra.

No.	MRR	Surface Roughness Ra
1	0.8198	0.0064
2	0.9361	0
3	1	0.96702
4	0.2836	0.7432
5	0.6314	1
6	0.6605	0.5269
7	0.3241	0.3034
8	0.5388	0.2516
9	0	0.0385

**Table 8:** The grey relational coefficients of the MRR and surface roughness Ra.

No.	MRR	Surface Roughness Ra
1	0.7351	0.3348
2	0.8866	0.3333
3	1.0000	0.9384
4	0.4111	0.6607
5	0.5756	1.0000
6	0.5956	0.5138
7	0.4252	0.4179
8	0.5202	0.4005
9	0.3333	0.3421

**Table 9:** The MPCI of each parameter combination.

No.	1	2	3	4	5	6	7	8	9
MPCI	0.524	0.580	0.864	0.532	0.754	0.559	0.443	0.454	0.392

**Table 10:** The experimental results of CMSP for the cemented carbide insert cutting edge.

No.	A, Polishing Plate’s Rotational Speed (rpm)	B, Swing Angle (°)	C, Input Frequency of the Controller (Hz)	Linear Edge Radius (μm)	Circular Edge Radius (μm)
1	30	6	7000	46.895	70.619
2	30	4	5000	34.508	54.862
3	30	2	3000	33.011	49.780
4	60	6	5000	71.259	80.616
5	60	4	3000	41.822	57.024
6	60	2	7000	60.273	74.764
7	90	6	3000	67.279	78.155
8	90	4	7000	46.938	73.451
9	90	2	5000	59.435	74.113

**Table 11:** The S/N ratio of the linear edge radius and circular edge radius.

No.	A, Polishing Plate’s Rotational Speed (rpm)	B, Swing Angle (°)	C, Input Frequency of the Controller (Hz)	SNR of the Linear Edge Radius (dB)	SNR of the Circular Edge Radius (dB)
1	30	6	7000	33.423	36.978
2	30	4	5000	30.758	34.785
3	30	2	3000	30.373	33.941
4	60	6	5000	37.057	38.128
5	60	4	3000	32.428	35.121
6	60	2	7000	35.602	37.474
7	90	6	3000	36.558	37.859
8	90	4	7000	33.430	37.320
9	90	2	5000	35.481	37.398

**Table 12:** ANOVA results of CMSP for the insert linear edge radius.

Source	Degree of Freedom	Sum of Squares	Mean Square	F Value	*p* Value	Contribution (%)
A	2	775.89	387.945	21.784	0.044	47.91
B	2	644.679	322.34	18.1	0.052	39.42
C	2	88.897	44.448	2.496	0.286	3.45
Error	2	35.617	17.809			9.22
Total	8	1545.083				100

Significant at 95% confidence interval.

**Table 13:** ANOVA results of CMSP for the insert circular edge radius.

Source	Degree of Freedom	Sum of Squares	Mean Square	F Value	*p* Value	Contribution (%)
A	2	455.878	227.939	73.068	0.014	44.66
B	2	340.29	170.145	54.542	0.018	33.18
C	2	204.399	102.199	32.761	0.03	19.68
Error	2	6.239	3.12			2.48
Total	8	1006.806				100

Significant at 95% confidence interval.

## Data Availability

The authors confirm that the data supporting the findings of this study are available within the article.
